# Behavioural Indicators of Intra- and Inter-Specific Competition: Sheep Co-Grazing with Guanaco in the Patagonian Steppe

**DOI:** 10.3390/ani11113333

**Published:** 2021-11-22

**Authors:** Tomás Fernández, Alex Lancaster, Claudio A. Moraga, Sergio Radic-Schilling, Achaz von Hardenberg, Paulo Corti

**Affiliations:** 1 Laboratorio de Manejo y Conservación de Vida Silvestre, Instituto de Ciencia Animal y Programa de Investigación Aplicada en Fauna Silvestre, Facultad de Ciencias Veterinarias, Universidad Austral de Chile, Valdivia 5090000, Chile; tomas.fv@gmail.com; 2 Programa de Magister en Ecología Aplicada, Instituto de Ciencias Ambientales y Evolutivas, Facultad de Ciencias, Universidad Austral de Chile, Valdivia 5090000, Chile; 3 Conservation Biology Research Group, Department of Biological Sciences, University of Chester, Chester CH1 4BJ, UK; 1821496@chester.ac.uk (A.L.); a.vonhardenberg@chester.ac.uk (A.v.H.); 4 Centro de Estudios del Cuaternario de Fuego-Patagonia y Antártica (Fundación CEQUA), Punta Arenas 6200000, Chile; clmoraga@gmail.com; 5 Department of Wildlife Ecology and Conservation, School of Natural Resources and the Environment, University of Florida, Gainesville, FL 32611, USA; 6 Departamento de Ciencias Agropecuarias y Acuícolas, Facultad de Ciencias, Universidad de Magallanes, Punta Arenas 6200000, Chile; sergio.radic@umag.cl

**Keywords:** density, feeding behaviour, bite rate, intraspecific competition, interspecific competition, guanaco, sheep

## Abstract

**Simple Summary:**

In extensive livestock ranching, where animals are maintained at high numbers, competition between individuals is expected, but not generally assumed. To compensate for reduced food availability, herbivores modify their feeding behaviour, which can be used as an indicator of competition. We investigated behavioural changes of domestic sheep in Chilean Patagonia in response to herd size, food availability, and the presence of a wild competitor, the guanaco, considered a problem for sheep production by ranchers. Large sheep herds were associated with a decrease in sheep grazing, while food availability increased time spent feeding. Guanaco had no effect on sheep behaviours. Behavioural changes were mostly associated with competition between individual sheep and not with guanaco. We suggest that to improve sheep production, ranchers should focus on sheep management at appropriate herd sizes according to grassland capacities.

**Abstract:**

In extensive livestock production, high densities may inhibit regulation processes, maintaining high levels of intraspecific competition over time. During competition, individuals typically modify their behaviours, particularly feeding and bite rates, which can therefore be used as indicators of competition. Over eight consecutive seasons, we investigated if variation in herd density, food availability, and the presence of a potential competitor, the guanaco (*Lama guanicoe*), was related with behavioural changes in domestic sheep in Chilean Patagonia. Focal sampling, instantaneous scan sampling, measures of bite and movement rates were used to quantify behavioural changes in domestic sheep. We found that food availability increased time spent feeding, while herd density was associated with an increase in vigilant behaviour and a decrease in bite rate, but only when food availability was low. Guanaco presence appeared to have no impact on sheep behaviour. Our results suggest that the observed behavioural changes in domestic sheep are more likely due to intraspecific competition rather than interspecific competition. Consideration of intraspecific competition where guanaco and sheep co-graze on pastures could allow management strategies to focus on herd density, according to rangeland carrying capacity.

## 1. Introduction

In free-ranging herbivores, intraspecific competition increases with population density when food resources become limited [1]. Increased competition for limited resources has been shown to delay the age of first reproduction [2], increase new-born mortality [3,4,5], as well as juvenile and adult mortality rates [5,6]. Consequently, an increase in the population density, above the carrying capacity, typically triggers density-dependent processes, leading to a reduction in the population [7,8].

Herbivores may modify their feeding behaviour in response to an increase in competition for resources [9,10]. Individuals may adjust both time spent feeding and resource intake intensity as a mechanism to compensate for lower food availability to satisfy their nutritional requirements [10,11]. For example, American bison (*Bison bison*), during winter, spend more time feeding in higher quality patches when the group size is bigger [12]. Elk (*Cervus canadensis*) [13], Alpine ibex (*Capra ibex*) [14], and South African oryx (*Oryx gazella*) [15] increase their food intake rate when food resources are scarce.

Contrary to wild herbivores, domestic herbivores are usually spatially confined, with population densities determined by productive interests rather than environmental constraints, predisposing them to increased intraspecific competition if trophic resources are limited [16,17]. In gregarious wild herbivores, when intraspecific competition increases, individuals tend to move away or split into smaller subgroups to avoid and decrease competition, but these mechanisms may fail if densities are artificially maintained at high levels [17,18].

Similar to wild herbivores, domestic herbivores also modify their feeding behaviour when intraspecific competition increases [9]. In domestic goats and cattle, an increase in the number of feeding competitors increased the food intake rate [19,20,21]. In domestic sheep, an increase in the population density and a lower food availability increased the time individuals spent feeding daily [22,23].

Intraspecific competition in livestock might be exacerbated by the competition of wild herbivores grazing on the same grounds [24], and there is evidence of the negative impacts that wild herbivore grazing can have on cattle while competing for the same resources [25,26]. It has also been shown that interspecific competition can modify the feeding behaviour of the competing herbivores, e.g., decreasing their bite rate while increasing vigilance behaviours [27], or increasing the search time for resources [26]. 

In Patagonia, the increase and expansion of guanaco (*Lama guanicoe*) populations from protected areas to livestock grasslands has resulted in an increase in the co-occurrence with domestic sheep [28]. In addition, sheep ranching has steadily decreased in recent decades, reducing sheep numbers across Patagonia [29], possibly contributing to the expansion of guanaco into ranches. Several studies have pointed out a high diet overlap between these species and how this might contribute to resource competition [30,31,32]. Traba et al. [33] have shown a reduction in sheep’s spatial niche in the presence of guanaco during winter. However, Pontigo et al. [32] demonstrated that sheep do not modify their trophic niche in the presence of guanaco during summer, whereas guanaco do.

Ranchers claim that the increase in guanaco populations has a negative impact on livestock yield [34]. Additionally, some authors have suggested that the increase in the size of guanaco populations may be responsible for the overgrazing of the steppe in Argentinian Patagonia, reducing the available resources [35]. Marino et al. [36] suggested instead that the current overgrazed rangeland is caused by unsustainable domestic sheep population densities through the years, reaching as much as 73% above the carrying capacity in some areas of Argentinian Patagonia. Additionally, in Chilean Patagonia, it is assumed that some parts of the region exceed the carrying capacity of the steppe [37].

The livestock production system in Patagonia has remained largely similar for several decades, maintaining relatively consistent densities throughout the years [36,38,39]. The maintenance of animal densities above the steppe grassland carrying capacity, over long periods of time, is rarely seen in wild species under natural conditions [3,40]. Therefore, the current circumstances in sheep ranching in southern Patagonia offer a unique opportunity to observe the behavioural effects of intraspecific competition, in the presence of another species as a possible competitor [22,23,26,27].

Our objective was to evaluate intraspecific and interspecific competition affecting domestic sheep through changes in sheep feeding behaviours according to food availability, population density, and the co-occurrence of a wild herbivore species, the guanaco. We assessed variations in time spent feeding and resource intake intensity, through bite rates as indicators of intraspecific competition [20,22,23]. We compared the changes in behaviour between sites with and without guanaco presence to test for the effect of interspecific competition [41]. Our predictions were: (1) sheep increase both their feeding intake and time spent feeding as trophic resources decrease when population density is high [20,22,23], and (2) the presence of guanaco decreases sheep bite rate due to an increase in vigilance behaviour [27] and displacement [26].

## 2. Materials and Methods

### 2.1. Study Sites

**Figure 1:**
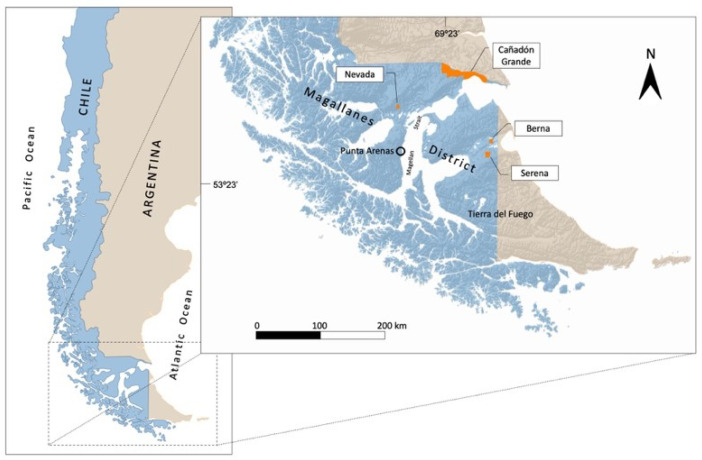
Location of study sites in Magallanes region. Sites are in Chilean Patagonia, with Cañadón Grande and Nevada ranches located on the mainland, and Berna and Serena ranches on the island of Tierra del Fuego.

The study was carried out on four sheep ranches in the Magallanes district of Chilean Patagonia (Figure 1), where sheep have been present since the late 19th Century [42]: Cañadón Grande (ca. 72,000 ha; 52°11′ S, 69°14′ O) and Nevada (ca. 1200 ha; 52°38′ S, 70°52′ O) on the mainland, and Serena (ca. 4600 ha; 53°21′ S, 68°53′ O) and Berna (ca. 2400 ha; 53°09′ S, 68°47′ O) on the Island of Tierra del Fuego. With the exception of Nevada, all ranches use continuous grazing systems, switching between winter and summer grasslands. This practice allows the grassland to recover from the previous grazing season [43]. The Nevada ranch uses a rotational grazing system with shorter grazing periods on smaller grasslands [43,44]. In Cañadón Grande and Serena ranches, there was a constant presence of guanaco in sheep grazing areas. Berna and Nevada ranches were used as control sites since guanacos were rarely, if ever, observed in grazing areas. The herds in all ranches were mainly composed of ewes, with reproductive activity onset at late summer and early autumn (rut season), lambing one or two individuals between late winter and early spring [45].

Potential predators of sheep and guanacos on mainland ranches are puma (*Puma concolor*), culpeo (*Lycalopex culapeus*) and grey (*L. griseous*) foxes [46]. Pumas predate on adults and young animals, but foxes mostly opportunistically prey on new-borns. Puma are absent from Tierra del Fuego Island, but both fox species are present [47]. In addition, domestic dog attacks on sheep and guanaco are increasing across Patagonia [48]. Raptors such as buzzard eagle (*Geranoaetus melanoleucus*) and southern caracara (*Caracara plancus*) might predate on new-born lambs [49].

Magallanes has a semiarid-cold climate, with four seasons [50]. Temperatures varied between a mean of 10.6 °C in summer (December–February) and a mean of 2.2 °C in winter (June–August), with precipitation ranging from a maximum monthly mean of 39.6 mm (April–July) and a minimum of 19.8 mm (September–October), averaging 358 mm annually with 25 mm of snow [51,52].

The Patagonian steppe grassland is dominated by graminoid species of tussock grasses (*Festuca gracillima*), needle grasses (*Stipa* spp.), meadow grasses (*Poa* spp.), and wallaby grass (*Rytidosperma* spp.), with a wide range of annual species growing between graminoids and transition areas associated with meadows and small and medium shrubs [53]. All sites are typical flat Patagonian steppe with some small rolling hills, but Nevada and Serena presented some areas with low shrubs on the side of the hills. Grassland primary productivity is variable, averaging between 455 and 2021 kg dry matter (dm)·ha^−1^·year^−1^, depending on the province in the district [54,55,56].

### 2.2. Food Seasonal Availability and Variation

Available dry vegetation biomass (kg dm·ha^−1^) was determined to quantify food availability in different areas for each ranch. Samples were collected through eight consecutive seasons from autumn 2018 to summer 2019 (year 1) and from autumn 2019 to summer 2020 (year 2). A total of 1636 vegetation samples were collected, varying between 32 and 66 samples per site/season. Between three to four line transects (ca. 2–5 km) were conducted on foot at each site per season, through different vegetation communities, randomly selecting three samples every 500 m with a 0.1 m^2^ Daubenmire quadrat [57] and collecting all aboveground vegetation within the quadrat [58]. Each sample was georeferenced with a GPS unit (Garmin 64s, Olathe, Kansas, USA), oven-dried at 60 °C for 48 h, and individually weighed [59]. Dry matter availability was estimated averaging samples from each site/season [59]. To account for grazed dry matter during the study, we estimated sheep consumption during the period between sampling seasons. Sheep daily intake ranges between 1.2 and 1.9 kg dm·day^−1^ depending mainly on food quality [60,61,62]. In Chilean Patagonia, sheep yearly intake has been estimated at around 650 kg dm·year^−1^ [63], therefore we estimate a daily intake of 1.78 kg dm·day^−1^. This value was multiplied by sheep density in each ranch, during the previous season of the dry matter sampling and considering an average of 90 days of consumption among seasons (three months between each fieldwork session). Steppe primary productivity for each ranch and season was then calculated by adding collected dry matter and estimated grazed dry matter [59,64]. Yearly primary productivity for each site and year was estimated as the mean of the seasonal productivities in the same year.

Carrying capacity was estimated annually for each site following Golluscio et al. [65] and Hashemi [66], dividing yearly primary productivity for each site by one Animal Unit Year (AUY) or ca. 4300 kg dm·year^−1^ [44,67,68,69]. Then, the carrying capacity was adjusted to the annual requirement of sheep, considering ca. 650 kg dm·year^−1^ [57] to calculate the Animal Unit Equivalent for sheep (AUE; 1 AUY = 0.15 AUE) [53,68,70]:

(1) $$\mathrm{AUE}=\frac{\mathrm{Annual}\;\mathrm{primary}\;\mathrm{productivity}\;\left(\mathrm{kg}\;\mathrm{dm}\cdot {\mathrm{ha}}^{-1}\cdot {\mathrm{year}}^{-1}\right)}{\mathrm{AUY}\;\left(4300\;\mathrm{kg}\;\mathrm{dm}\cdot {\mathrm{year}}^{-1}\right)}\cdot 6.67$$

The multiplying factor of 6.67 corresponds to the equivalent of 1 AUY = 0.15 AUE (1/0.15 = 6.67). Therefore, the carrying capacity indicates the number of AUE that the grassland can support each year.

### 2.3. Sheep Density, Stocking Rate and Guanaco Density

Sheep and guanaco densities were estimated for each site/season using the distance sampling method [71,72]. A total of 246.5 km was surveyed for sheep density estimation and 144 km for guanaco. At each site, 6–10 km line transects were conducted each season. Surveys at each site were completed in a single day to avoid recounting individuals who may have moved to other areas and overestimating by counting the same individuals on multiple occasions [73]. Group size was recorded, considering a group as the individuals within 50 m of each other and showing a coordinated movement [21,74]. Conventional (CDS) and multiple covariate distance sampling (MCDS) models were fitted to estimate the density for each species at each site, selecting the best model with Akaike Information Criterion (AIC), using the software Distance v.7.2 [72].

The stocking rate of sheep was defined as the Animal Units (AU) in a certain area during a certain season [44,67,68,69]. To compare stocking rate with rangeland carrying capacity, density was estimated as individuals·ha^−1^, and the annual mean was estimated for each year. Therefore, mean sheep density·ha^−1^·year^−1^ can be compared to AUE [67].

### 2.4. Sheep Behavioural Variation

#### 2.4.1. Group Activity Budgets

Activity budgets were estimated by directly observing randomly selected groups with the instant sampling method [75,76]. The group was observed from a minimum distance of 50 m using a scope (Nikon Prostaff 5 60x, Tokyo, Japan), to avoid influencing their behaviour, then sampling began when sheep returned to feeding. Sampling sessions were started once the observed animals started to forage again, after an observer arrived in the observation spot. Each individual behaviour in a group was recorded at the beginning of the sampling session and once every 5 min during the 15 min sample [15,77]. Recorded behaviours were defined as: (i) feeding: bites and extraction of vegetation, walking while maintaining the snout near the ground [22,78], (ii) vigilance: head lifted above the body to inspect surroundings without displacement [27,78], (iii) walking: individual walking with head lifted from the ground (to differentiate from feeding) [78,79], and (iv) other behaviours: including resting, fighting, maternal behaviour, reproductive behaviour, rumination, urination/defecation, fleeing, and grooming [79,80]. Behaviours classified as “other” were grouped due to their low observation frequency. From the 406 sheep groups, 1477 instant sampling records were obtained, making a total of 10,858 individual records. For activity budgets, the proportion of individuals feeding, vigilant, walking, and in other behaviours in each group was calculated from the total of individuals in the observed group. Approximately 46 groups were sampled in each season.

#### 2.4.2. Focal Observations

Focal observations were carried out to record changes in sheep bite and movement rates [75]. One randomly selected individual from each instantaneous scan sampled group was also observed, as described earlier. All behaviours displayed by the focal individual were recorded during a 15 min observation period [76]. Bites and steps were recorded during the 15 min observation period using handheld counters.

Bites were defined as the partial or complete extraction of vegetation from the ground with the mouth, followed by a quick, elevated jaw movement [27,81]. Steps were defined as the forward movement of either front limb [26]. Food intake rate was calculated by the number of bites taken while feeding (bites·min^−1^) and movement rate by the number of steps taken (steps·min^−1^) while feeding and walking [26,27,82]. To estimate the bite rate, 343 individuals were included, with a total of 4868 min of observations and a mean of 10.78 ± 2.5 focal individuals per site/season. For movement rate, 324 individuals were observed, adding up to 4663 min of observation and an average of 10.13 ± 2.6 focal individuals per site/season. All behaviours were recorded with the Animal Observer app [83] using an iPad mini tablet (iPad mini 4, Apple Inc., Cupertino, CA, USA).

### 2.5. Statistical Analysis

#### 2.5.1. Group Activity Budgets

Variations in group activity budgets were analysed in relation to food availability (kg dm·ha^−1^), sheep herd density (individuals·ha^−1^), group size, density of guanaco, study site, and season. Continuous variables (food availability, herd density, group size, and guanaco density) were standardised with a mean of 0 and standard deviation of 1 [84]. Generalised linear mixed models (GLMM) were fitted with a binomial error distribution and a logit link function, using group ID as a random variable [85,86].

#### 2.5.2. Focal Observations

Linear models (LM) were fitted to evaluate the relationship of food availability, sheep density, guanaco density, study site, and season with bites and step rates [87]. Step rate was normalised by exponential transformation [88], and all continuous variables were centred and standardised [84].

#### 2.5.3. Model Selection

Model selection was based on the AIC, considering the model with the lowest AIC value as the best fitting model and considering all models with a difference in AIC values from the best fitting model (ΔAIC) < 2 as equivalent [89,90]. If ΔAIC < 2, fulfilment of parsimony criteria was considered for model selection [86,91]. All statistical analyses were performed in R v.3.6.3 [92] using the lme4 [93] and the MuMIn [94] packages.

## 3. Results

### 3.1. Food Availability and Carrying Capacity

**Figure 2:**
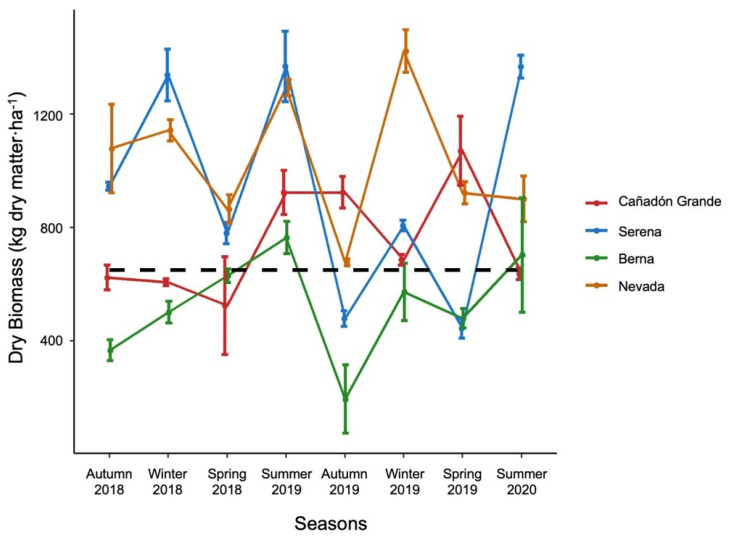
Dry matter productivity (kg dm·ha^−1^) at each study site per season. The black dashed line indicates necessary dry matter for 1 Animal Unit Equivalent (AUE; 650 kg dm·ha^−1^·year^−1^). Error bars indicate standard error.

Available dry biomass (kg dm·ha^−1^) varied between sites and seasons (Figure 2). The lowest annual average of food availability was registered in Cañadón Grande ranch with 564.32 ± 170.58 (SD) and 487.18 ± 215.34 kg·ha^−1^·year^−1^ in the first and second year of the study, respectively. Nevada recorded the highest available dry biomass, averaging 1146.85 ± 265.25 kg·ha^−1^·year^−1^ during the first year and 980.18 ± 314.48 in the second year. The yearly average for Serena was 1138.7 ± 328.01 in the first year and 773.51 ± 427.34 kg·ha^−1^·year^−1^ in the second year, while Cañadón Grande registered 669.16 ± 175.14 and 828.76 ± 203.79 kg·ha^−1^·year^−1^ each year. Carrying capacity estimated for each site, for the first and second year, were Berna: 0.88 and 0.76 AUE, Nevada: 1.70 and 1.52 AUE, Cañadón Grande: 1.04 and 1.29 AUE, and Serena: 1.72 and 1.20 AUE.

### 3.2. Sheep Density, Stocking Rate and Guanaco Density

There were 722 sheep groups recorded, for a total of 35,672 individuals, and 253 guanaco groups recorded, with a total of 1378 individuals. Mean animals per group were 49.3 for sheep and 5.4 for guanaco. Densities varied widely between sites and seasons, according to selected models (Appendix A, Table A1). Average sheep density (mean ± SD) was 1.82 ± 1.46 individuals·ha^−1^ throughout the study. Mean herd density for Berna and Nevada ranches was 1.43 ± 0.97 and 3.03 ± 2.12 individuals·ha^−1^ respectively, throughout the study. Cañadón Grande and Serena average herd densities were 1.8 ± 0.51 and 1.1 ± 1.2 individuals·ha^−1^, respectively. Guanacos mean density was 0.15 ± 0.17 individuals·ha^−1^, with an average of 0.24 ± 0.22 individuals·ha^−1^ in Cañadón Grande and 0.06 ± 0.03 individuals·ha^−1^ in Serena.

Stocking rate for each site for the first and second year was: Berna: 1.31 and 1.54 AUE·ha^−1^, Nevada: 3.46 and 2.59 AUE·ha^−1^, Cañadón Grande: 1.84 and 1.75 AUE·ha^−1^, and Serena: 1.63 and 0.58 AUE·ha^−1^. All sites registered stocking rates above their carrying capacity in both years, meaning the rangelands were overgrazed, except for Serena which featured a slightly lower stocking rate than its carrying capacity during the first year, and below half during the second year.

### 3.3. Sheep Behaviour Variation

#### 3.3.1. Groups’ Activity Budgets

**Figure 3:**
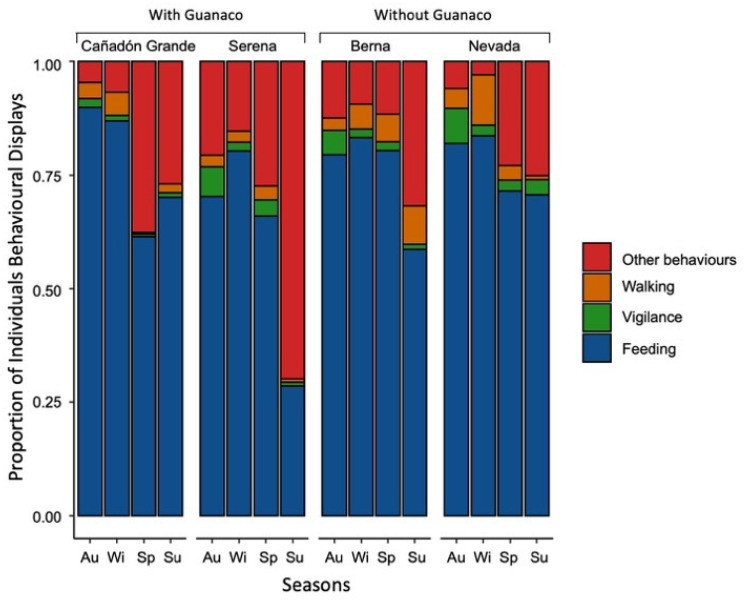
The proportion of individuals in groups displaying feeding, walking, vigilance, and other behaviours for each study site and season.

Activity budgets varied significantly between sites and seasons (Figure 3). Feeding behaviour represented 73.54% of all behaviours in the groups, followed by “other behaviours” (20.13%), walking (3.91%), and vigilance (2.41%). Selected models showed that the main variables affecting the behaviours were sheep density, group size, and season, besides interactions of density with group size, season with group size, and feeding behaviour with food availability (Table 1).

**Figure 4:**
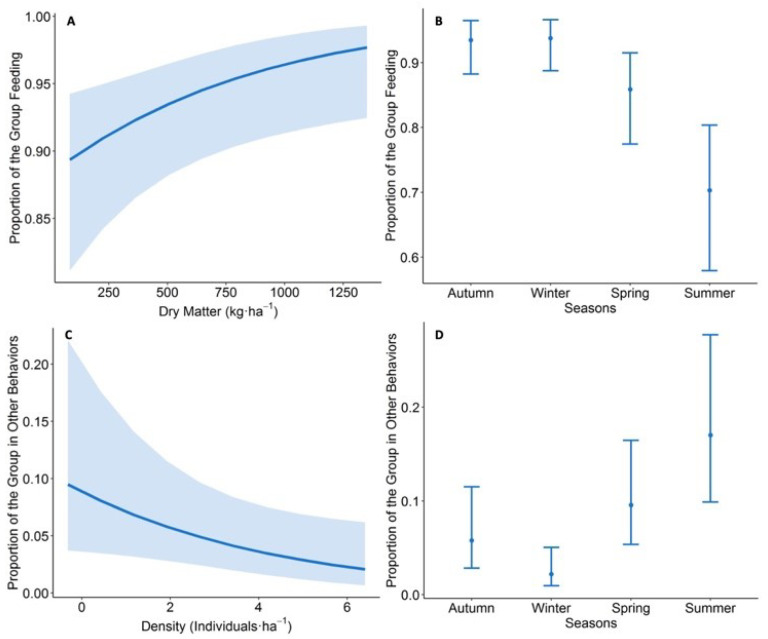
The effect of food availability, herd density, and season on sheep behaviour, according to selected models. (**A**) Effect of dry matter on the proportion of the group feeding. (**B**) Variation in the proportion of the group feeding in different seasons. (**C**) Effect of sheep density on the expression of other behaviours. (**D**) Expression of other behaviours across different seasons. Light-blue shadows and bars indicate 95% confidence intervals.

Food availability had a positive relationship with the proportion of individuals feeding (Figure 4A; Appendix A, Table A2). In spring and summer, there was a lower proportion of individuals feeding in observed groups compared to autumn (Figure 4B; Appendix A, Table A2). Group size had no relationship with the proportion of individuals feeding, except during summer, when a lower proportion of sheep was observed feeding. Vigilant behaviour increased together with density but decreased when group size increased (Appendix A, Table A2). Walking behaviour increased in winter, compared to autumn (Appendix A, Table A2).

The display of other behaviours was negatively related with sheep density (Figure 4C; Appendix A, Table A2). The proportion of other behaviours was lower in winter and higher in summer (Figure 4D), when compared to autumn. Group size was positively related with the other behaviours, but only in spring and summer. During summer, an inverse relation was observed between the effect of group size on feeding behaviour and on other behaviours.

#### 3.3.2. Bite Rate

**Figure 5:**
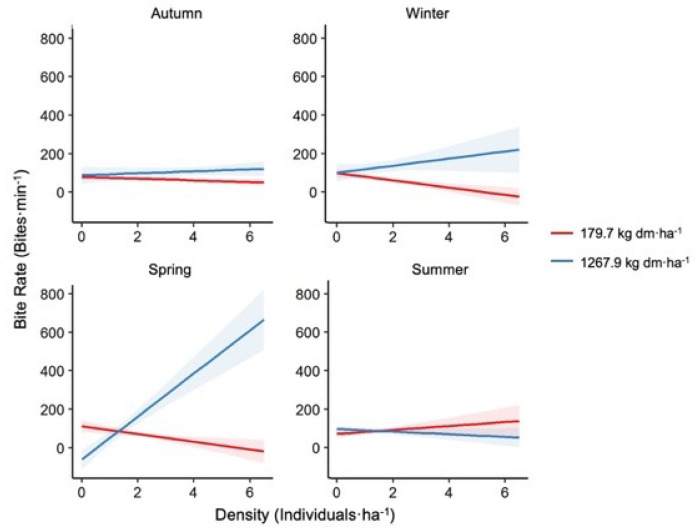
Variation of bite rate (bites·min^−1^). Bite rate values according to herd density in each season, in conditions of lower food availability (red) and high food availability (blue). Shadowed areas indicate 95% confidence intervals.

The best fitting model included the interaction of density and food availability with different seasons, besides the effect of seasons on itself. (Table 2). Mean bites·min^−1^ was 102.99 ± 23.12, ranging from 36.13 to a maximum of 171.07 bites·min^−1^. Bites·min^−1^ varied between seasons and was comparatively higher in spring than autumn (Appendix A, Table A3). Sites registered different bites·min^−1^, being higher in Berna and Nevada than Cañadón Grande and Serena ranches. Dry biomass availability was positively related with bite rate during winter and spring, but not in autumn and summer. Sheep density had different effects on bite rate according to the season. During spring, density was positively related with bite rate, while it was negatively related during winter. Interactions between herd density, dry biomass available, and season showed a negative effect of density on the bite rate of sheep, while food available was lower during winter and spring (Figure 5), but a positive effect of food availability was higher during the same seasons.

#### 3.3.3. Movement Rate

Movement rate had a mean of 10.85 ± 6.21 steps·min^−1^, with a minimum of 1.47 and maximum of 29.94 steps·min^−1^. The selected model showed higher movement rates in spring and summer compared to autumn (Appendix A, Table A3). Food availability was positively related with movement rate, but only during winter.

## 4. Discussion

To our knowledge, this is the first empirical study analysing intraspecific competition in sheep through behavioural changes in Patagonia. We showed an effect of dry biomass availability and sheep density on feeding behaviour and bite rate, as well as an effect of sheep density on vigilance and other behaviours. Our results suggest that the current high densities of sheep herds above carrying capacity in some sites increase intraspecific competition, leading to changes in sheep feeding behaviour [18,20,22,23]. Although the presence of a possible competitor species could potentially increase these negative effects due to interspecific competition [26,41,82], we found no evidence of any negative effect caused by the presence of guanaco co-grazing with sheep in the sites under study.

Behavioural changes observed in sheep were expected, with stocking rates above the carrying capacity in almost every site, as seen in other species [22,23]. At low densities, low food availability increased the bite rate, which is possibly related to a process of compensation [13,14]. As density increased, bite rate in areas and seasons with higher food resource availability also increased. In accordance with Shrader et al. [20] and Chen et al. [95], the increase in bite rate in domestic sheep and goats, as group size increases, could act as a mechanism to exploit resources before their competitors can and may compensate for any negative effect caused by competition. As with sheep in our study, Odadi and Rubenstein [96] also showed an increase in the bite rate of cattle when grazing in larger groups with restricted access to grazing areas. This strategy could be a response to a resource exploitation competition process, where individuals aim to use the resources before their competitors [82,97]. Interference competition affects the intake of resources and leads to the expression of other behaviours, such as vigilance, displacement, or agonistic behaviour [98,99], as has been shown in several ungulate species, such as mule deer (*Odocoileus hemionus*) and red deer (*Cervus elaphus*) [100], or impala (*Aepyceros melampus*), zebra (*Equus quagga*), and kudu (*Tragelaphus strepsiceros*) [101]. Similarly, herd density in areas and seasons with low food availability had a negative impact on bite rate and increased vigilant behaviours in sheep within our study. 

The positive effect of food availability on feeding behaviour was contrary to our expectations, as low food resources should increase both bite rate and time spent feeding as a mechanism to compensate for resource scarcity [9,23,95,102]. Bergman et al. [11,103] showed that bison have a time-minimizing foraging strategy that allows them to increase time spent feeding when food resources are more abundant and nutritional requirements are higher. This could be the same strategy used by sheep in Patagonia, increasing their time feeding due to higher nutritional requirements, thus exploiting every available resource. In addition, it has been shown that domestic livestock on low-quality forages present larger movements, tending to forage more selectively [104].

The higher proportion of individuals feeding, and lower proportion of other behaviours expressed, is possibly not only related to food availability, but also to daylight periods and weather conditions in winter, such as lower temperatures and higher levels of precipitation [13,21,22,105]. Studies show that reduced feeding time due to fewer daylight hours in winter or experimental restricted access to pastures, increase the proportion of time spent feeding and reduce the time spent in other activities, related to the total time available to feed [22,106,107,108]. Furthermore, in winter, ungulates use lower-quality patches [105,109,110] and the resource encounter rate is reduced due to changes in the environment, such as the presence of snow on the terrain [13], eventually leading to an increase in searching and therefore feeding times.

Guanaco co-grazing with sheep had no significant effect on the time allocated to feeding or bite rates in sheep. None of the best fitting models included guanaco density as a significant variable. Guanaco densities on sheep grazing areas are possibly not high enough to have a measurable impact on them [111,112]. A lack of interspecific competition has also been reported for sheep co-grazing with other species, such as red deer [113] and chamois (*Rupicapra rupicapra*) [114]. However, sheep presence seemed to reduce the trophic niche of guanaco when co-occurring [32]. Chesson [115] proposed that two species can coexist exploiting the same resources, as long as the effect of intraspecific competition is stronger than interspecific competition, as may be occurring between sheep and guanaco in Patagonia. Guanaco presence did not increase sheep vigilance, walking, aggression or other behaviours that may interrupt resource intake.

## 5. Conclusions

Our results suggest that behavioural changes in sheep grazing the Patagonian steppe are related to intraspecific competition, rather than interspecific competition with sympatric wild guanaco. Competition intensity is influenced by herd density, which is maintained at stocking rates above that of carrying capacities in some rangeland sites on the Patagonian steppe. This finding should be considered an important point of concern because this ranching system will likely not be sustainable in the mid- to long-term. 

In situ evaluation of sheep behaviour may be applied as an indicator of increases in intraspecific competition, allowing timely changes to management strategies. Schönbach et al. [116] showed that a reduction in stocking rates can maintain economic benefits while applying sustainable grassland management. Briske et al. [117] proposed a plan to modify an inner Mongolia grazing system, focused on a market-based production system, supported by developing livestock raising technification, lower stocking rates, and high-quality products, allowing to maintain profits while reducing overgrazing impacts on pastures. Similar actions could be applied in southern Patagonian sheep ranching, improving livestock productivity and sheep welfare, reducing overgrazing negative impacts on the steppe.

## Figures and Tables

**Table 1:** Generalised linear mixed models (GLMM) fitted for feeding, vigilance, walking, and other behaviours. Bold indicates selected model. Variables correspond to available dry biomass (kg dm·ha^−1^; biomass), group size (group.size), sheep density (ds.oa), guanaco density (ds.lg), seasons (seasons), and study site (site). Random variable Scan.id corresponds to the evaluated group. Models’ degrees of freedom (DF), Akaike Information Criterion value (AIC), difference in AIC values between each model to lower AIC model (ΔAIC), AIC weight (AICω), and conditional χ^2^ (R^2^c) are shown.

Model	DF	AIC	ΔAIC	AICω	R^2^c
Feeding					
biomass + group.size *seasons + site + (1|scan.id)	**13**	**1261.10**	**0.00**	**0.413**	**0.382**
biomass + ds.oa + group.size *seasons + site + (1|scan.id)	14	1261.88	0.78	0.280	0.383
biomass *group.size + group.size *seasons + site + (1|scan.id)	14	1263.10	2.00	0.152	0.382
group.size *seasons + site + (1|scan.id)	12	1264.95	3.85	0.060	0.379
group.size *seasons + (1|scan.id)	9	1265.82	4.72	0.039	0.375
biomass *group.size + seasons + site + (1|scan.id)	11	1265.88	4.78	0.038	0.378
Vigilance					
**ds.oa + group.size + ds.oa:group.size + (1|scan.id)**	**5**	**165.42**	**0.00**	**0.379**	**0.685**
biomass + ds.oa + group.size + (1|scan.id)	5	165.57	0.15	0.351	0.459
ds.oa + group.size + (1|scan.id)	4	167.56	2.14	0.130	0.412
ds.oa + group.size + ds.lg + (1|scan.id)	5	168.21	2.79	0.094	0.442
biomass + ds.oa + group.size + seasons + (1|scan.id)	8	170.34	4.93	0.032	0.491
biomass + ds.oa + group.size + season + ds.lg + (1|scan.id)	9	172.00	6.58	0.014	0.465
Walking					
**seasons + (1|scan.id)**	**5**	**300.07**	**0.00**	**0.396**	**0.109**
biomass + seasons + (1|scan.id),	6	300.38	0.31	0.338	0.123
biomass + group.size + seasons + (1|scan.id),	7	301.99	1.92	0.151	0.127
biomass + group.size + ds.oa + seasons + (1|scan.id),	8	303.65	3.58	0.066	0.130
biomass + group.size + ds.oa + ds.lg + seasons + (1|scan.id),	9	305.46	5.39	0.027	0.132
biomass:seasons + seasons + (1|scan.id),	9	305.93	5.86	0.021	0.129
Other behaviours					
**ds.oa + group.size:seasons + seasons + site + (1|scan.id)**	**13**	**909.25**	**0.00**	**0.357**	**0.471**
ds.oa:seasons + group.size:seasons + seasons + site + (1|scan.id)	16	910.58	1.32	0.184	0.473
biomass + ds.oa + group.size:seasons + seasons + site + (1|scan.id)	14	910.60	1.35	0.182	0.470
group.size:seasons + seasons + site + (1|scan.id)	12	911.40	2.14	0.122	0.469
ds.lg + group.size:seasons + seasons + site + (1|scan.id)	13	912.39	3.13	0.074	0.471
biomass + group.size:seasons + seasons + site + (1|scan.id)	13	912.68	3.43	0.064	0.468

**Table 2:** Linear models (LM) for bite rate (bites·min^−1^) and movement rate (steps·min^−1^). Bold indicates selected model. Variables correspond to kg dm·ha^−1^ (biomass), group size (group.size), sheep density (ds.oa), guanaco density (ds.lg), seasons (seasons), and study sites (site). Models’ degrees of freedom (DF), Akaike Information Criterion (AIC), difference in AIC values between each model to lower AIC model (ΔAIC), and χ^2^-adjusted value (R^2^) are indicated.

Model	DF	AIC	ΔAIC	R^2^
Bite rate				
ds.oa:biomass + ds.oa:seasons + biomass:seasons + ds.oa:biomass:seasons + seasons + site	18	3143.88	0	0.22
ds.oa:biomass + ds.oa:seasons + biomass:seasons + ds.oa:biomass:seasons + seasons + site + ds.lg	19	3145.04	1.16	0.22
**ds.oa:seasons + biomass:seasons + ds.oa:biomass:seasons + seasons + site**	**20**	**3145.53**	**1.65**	**0.22**
ds.oa:seasons + biomass:seasons + ds.oa:biomass:seasons + seasons + site + ds.lg	21	3147.29	3.41	0.28
biomass:seasons + ds.oa:biomass:seasons + seasons + site	16	3165.61	21.73	0.16
ds.oa:biomass + ds.oa:seasons + biomass:seasons + ds.oa:biomass:seasons + seasons + ds.lg	16	3167.37	23.49	0.16
Movement rate				
**biomass:seasons + seasons**	**9**	**1994.57**	**0**	**0.10**
biomass:seasons + biomass:site + seasons	12	1998.13	3.56	0.10
biomass:seasons + site + seasons	12	2000.43	5.86	0.09
biomass:site + biomass:season	9	2004.35	9.78	0.07
biomass:site + biomass:seasons + site + seasons	15	2002.48	7.91	0.09
biomass:seasons + site	9	2007.47	12.90	0.06

## Data Availability

The data supporting the results and conclusions of this article will be made available by the authors upon reasonable request.

## Appendix A

**Table A1:** Sheep and guanaco density estimation for each site and season. Density of sheep or guanaco (individuals·ha^−1^), 95% confidence interval (95% CI), coefficient of variation (CV), model degrees of freedom (DF), and number of individuals (*n*).

**Sheep**	**Season**	**Density**	**95% CI**	**CV**	**DF**	***n***
Site						
Cañadón Grande (With guanaco)	Autumn 2018	0.84	0.15–4.66	74.28	4.90	1680
	Winter 2018	2.49	1.32–4.67	32.38	59.17	4975
	Spring 2018	1.83	0.93–3.54	32.16	16.39	3653
	Summer 2019	0.09	0.02–0.30	54.57	7.53	186
	Autumn 2019	0.97	0.61–1.53	22.16	19.91	1950
	Winter 2019	1.69	0.48–5.89	49.06	4.36	3387
	Spring 2019	2.91	0.84–10.0	61.47	11.84	5819
	Summer 2020	0.59	0.05–6.23	70.15	2.37	1187
Nevada (Without guanaco)	Autumn 2018	5.69	1.18–27.3	72.76	6.44	3953
	Winter 2018	3.02	0.99–9.14	54.74	12.73	2099
	Spring 2018	2.29	0.26–19.4	125.77	11.13	1592
	Summer 2019	2.85	1.03–7.81	52.12	25.59	1981
	Autumn 2019	6.49	1.86–22.6	64.58	16.58	4512
	Winter 2019	1.05	0.33–3.27	54.22	9.44	727
	Spring 2019	0.24	0.07–0.77	54.59	8.51	168
	Summer 2020	2.59	0.50–13.2	84.97	10.58	1799
Berna (Without guanaco)	Autumn 2018	2.30	1.10–4.76	31.13	6.54	6163
	Winter 2018	1.07	0.53–2.15	35.48	41.39	1788
	Spring 2018	2.28	1.04–4.98	33.81	6.83	6125
	Summer 2019	1.72	0.94–3.13	29.77	25.54	4615
	Autumn 2019	1.08	0.19–6.15	80.63	5.94	1806
	Winter 2019	2.31	0.85–6.22	48.35	12.57	3286
	Spring 2019	1.93	1.34–2.77	17.98	35.68	2752
	Summer 2020	1.67	0.81–3.39	33.60	12.40	4473
Serena (With guanaco)	Autumn 2018	3.38	1.06–10.6	55.42	10.06	6073
	Winter 2018	1.96	0.64–5.98	50.77	7.59	3527
	Spring 2018	1.12	0.41–3.02	49.03	13.74	2006
	Summer 2019	0.04	0.00–0.50	97.84	3.07	69
	Autumn 2019	0.29	0.05–1.45	82.08	9.43	520
	Winter 2019	0.24	0.06–0.92	56.70	5.22	434
	Spring 2019	0.61	0.09–3.84	84.17	5.41	1098
	Summer 2020	1.16	0.26–5.10	63.02	5.03	2081
**Guanacos**	**Season**	**Density**	**95% CI**	**CV**	**DF**	***n***
Site						
Cañadón Grande	Autumn 2018	0.12	0.03–0.41	50.25	4.61	316
	Winter 2018	0.19	0.04–0.78	57.57	4.33	310
	Spring 2018	0.15	0.04–0.56	56.34	5.52	407
	Summer 2019	0.02	0.00–0.03	26.86	6.07	43
	Autumn 2019	0.71	0.14–3.53	65.37	4.37	1186
	Winter 2019	0.33	0.15–0.67	29.12	5.33	467
	Spring 2019	0.32	0.14–0.71	37.02	8.88	450
	Summer 2020	0.05	0.01–0.15	43.60	4.96	141
Serena	Autumn 2018	0.09	0.03–0.21	40.56	9.65	161
	Winter 2018	0.09	0.04–0.18	33.52	18.35	166
	Spring 2018	0.01	0.00–0.02	38.10	7.29	19
	Summer 2019	0.06	0.01–0.16	52.81	9.07	99
	Autumn 2019	0.03	0.00–0.11	55.95	6.11	58
	Winter 2019	0.06	0.01–0.22	47.41	3.84	111
	Spring 2019	0.05	0.02–0.12	35.16	5.32	96
	Summer 2020	0.06	0.01–0.22	65.70	8.41	104

**Table A2:** Coefficients for fixed effects estimated for selected behavioural generalised linear mixed models. Variables correspond to kg dm·ha^−1^ (biomass), sheep density (ds.oa), group size (group.size), seasons (seasons), and study sites (site).

Model	Estimate	Std. Error	z Value	Pr (>|z|)
Feeding				
(Intercept)	2.665	0.332	8.031	<0.0001
biomass	0.358	0.149	2.410	0.0159
seasonsWinter	0.046	0.317	0.145	0.8850
seasonsSpring	−0.861	0.293	−2.933	0.0034
seasonsSummer	−1.802	0.343	−5.261	<0.0001
siteSerena	−1.178	0.357	−3.298	0.0010
siteBerna	0.029	0.303	0.095	0.9243
siteNevada	−0.468	0.313	−1.496	0.1346
group.size:seasonsAutumn	0.148	0.294	0.503	0.6151
group.size:seasonsWinter	−0.043	0.201	−0.211	0.8329
group.size:seasonsSpring	−0.184	0.176	−1.045	0.2960
group.size:seasonsSummer	−0.930	0.208	−4.468	<0.0001
Vigilance				
(Intercept)	−5.567	0.665	−8.372	<0.0001
ds.oa	0.953	0.298	3.203	0.0014
group.size	−2.175	0.918	−2.370	0.0178
ds.oa:group.size	1.011	0.477	2.120	0.0340
Walking				
(Intercept)	−3.978	0.412	−9.659	<0.0001
seasonsWinter	0.987	0.485	2.034	0.0419
seasonsSpring	−0.768	0.711	−1.080	0.2802
seasonsSummer	0.196	0.562	0.348	0.7280
Other Behaviours				
(Intercept)	−2.788	0.382	−7.300	<0.0001
ds.oa	−0.354	0.175	−2.025	0.0429
seasonsWinter	−1.004	0.436	−2.306	0.0211
seasonsSpring	0.541	0.361	1.499	0.1340
seasonsSummer	1.204	0.372	3.240	0.0013
siteSerena	0.5970	0.364	1.566	0.1174
siteCañadón Grande	−0.225	0.362	−0.621	0.5344
siteNevada	−0.010	0.383	−0.026	0.9790
group.size:seasonsAutumn	−0.178	0.335	−0.532	0.5944
group.size:seasonsWinter	0.378	0.252	1.496	0.1346
group.size:seasonsSpring	0.492	0.192	2.561	0.0104
group.size:seasonsSummer	1.147	0.232	4.952	<0.0001

**Table A3:** Coefficients estimated for selected bite and movement rate linear models. Variables correspond to kg dm·ha^−1^ (biomass), sheep density (ds.oa), seasons (seasons), and study sites (site).

Model	Estimate	Std. Error	t Value	Pr (>|t|)
Bite rate				
(Intercept)	79.295	3.998	19.832	<0.0001
seasonsWinter	8.450	4.402	1.920	0.0558
seasonsSpring	19.267	5.096	3.781	0.0002
seasonsSummer	11.63	6.013	1.934	0.0540
siteSerena	−3.252	5.749	−0.566	0.5720
siteBerna	21.244	4.784	4.440	<0.0001
siteNevada	21.025	4.879	4.309	<0.0001
ds.oa:seasonsAutumn	−2.118	2.304	−0.919	0.3587
ds.oa:seasonsWinter	−10.834	4.978	−2.176	0.0302
ds.oa:seasonsSpring	31.065	6.832	4.547	<0.0001
ds.oa:seasonsSummer	7.375	7.279	1.013	0.3117
seasonsAutumn:biomass	7.375	5.062	1.457	0.1461
seasonsWinter:biomass	19.816	4.870	4.069	<0.0001
seasonsSpring:biomass	21.632	6.217	3.479	0.0006
seasonsSummer:biomass	−1.936	3.427	−0.565	0.5726
ds.oa:seasonsAutumn:biomass	3.832	2.758	1.389	0.1657
ds.oa:seasonsWinter:biomass	14.918	6.181	2.414	0.0163
ds.oa:seasonsSpring:biomass	52.767	7.975	6.617	<0.0001
ds.oa:seasonsSummer:biomass	−6.892	4.319	−1.596	0.1115
Movement rate				
(Intercept)	17.086	0.609	28.055	<0.0001
seasonsWinter	0.417	0.873	0.478	0.6333
seasonsSpring	2.553	0.924	2.762	0.0061
seasonsSummer	2.767	0.945	2.929	0.0037
biomass:seasonsAutumn	−0.589	0.792	−0.743	0.4583
biomass:seasonsWinter	3.622	0.857	4.226	<0.0001
biomass:seasonsSpring	0.165	1.038	0.159	0.8737
biomass:seasonsSummer	−0.016	0.458	−0.035	0.9722
